# Supplementary material for: The Impact of Time between Booster Doses on Humoral Immune Response in Solid Organ Transplant Recipients Vaccinated with BNT162b2 Vaccines
Source: Viruses. 2024 May 28;16(6):860. doi: 10.3390/v16060860 (PMC11209529; doi:10.3390/v16060860)
Supplement: Supplementary file 1 [file viruses-16-00860-s001.zip › supplementary table 1.pdf]

| Supplementary table 1 | | | Rate ratio of monthly fold change in GMC between per one month increase in time interval between last two BNT162b2 vaccine doses | | |
|---|---|---|---|---|---|
| | **Fold change in GMC per month** | **95% CI** | **Rate Ratio** | **95% CI** | **p - value** |
| All | n = 107 | | | | |
| Crude | 1.13 | 0.87 – 1.46 | 1.04 | 1.01 – 1.08 | 0.021 |
| Adjusted | 0.95 | 0.71-1.27 | 1.04 | 1.01 – 1.08 | 0.022 |
| No infection during Follow-up | n = 90 | | | | |
| Crude | 0.79 | 0.62-1.02 | 0.78 | 1.05-1.09 | 0.001 |
| Adjusted | 0.80 | 0.62-1.04 | 1.05 | 1.02 – 1.08 | 0.001 |

Adjusted for age, sex, transplant type, time of last SARS-CoV-2 infection and monoclonal SARS-CoV-2 antibody therapy within six months of baseline, as appropriate.
