# Supplementary material for: The Impact of Time between Booster Doses on Humoral Immune Response in Solid Organ Transplant Recipients Vaccinated with BNT162b2 Vaccines
Source: Viruses. 2024 May 28;16(6):860. doi: 10.3390/v16060860 (PMC11209529; doi:10.3390/v16060860)
Supplement: Supplementary file 1 [file viruses-16-00860-s001.zip › supplementary table 2.pdf]

Supplementary table 2	**p – value for interaction between maintenance immunosuppresive therapy and interval between last two BNT162b2 doses when including all participants**	**p – value for interaction between maintenance immunosuppresive therapy and interval between last two BNT162b2 doses when excluding participants with SARS-CoV-2 infection between baseline and follow-up**
Corticosteroid (yes/no)		
Crude	0.768	0.822
Adjusted	0.810	0.911
CNI (yes/no)		
Crude	0.248	0.294
Adjusted	0.251	0.293
Antimetabolite (yes/no)		
Crude	0.239	0.987
Adjusted	0.328	0.769
